# Efficacy of long-acting growth hormone in Axenfeld-Rieger syndrome with a novel 3.824 Mb 4q25 deletion: a Case Report and systematic literature review

**DOI:** 10.3389/fgene.2026.1856235

**Published:** 2026-06-17

**Authors:** Shihui Guan, Jinyu Wang, Zhenxian Liu, Yangfan Qi, Xiaoyu Sun, Kai Jiang, Shuangzhu Lin

**Affiliations:** 1 School of Traditional Chinese Medicine, Changchun University of Chinese Medicine, Changchun, China; 2 Diagnosis and Treatment Center for Children, The Affiliated Hospital of Changchun University of Chinese Medicine, Changchun, China

**Keywords:** 4q25 microdeletion, axenfeld-rieger syndrome (ARS), genotype-phenotype correlation, growth hormone deficiency (GHD), longitudinal growth dynamics, *PITX2* gene

## Abstract

**Background:**

Axenfeld-Rieger syndrome (ARS) is a multisystem disorder primarily caused by *PITX2* mutations. While eye and dental anomalies are classic, the role of *PITX2* as a dosage-sensitive regulator of the human growth axis remains clinically under-recognized. We aim to elucidate the link between large-scale 4q25 microdeletions and severe growth hormone deficiency (GHD).

**Methods:**

A 5.4-year-old boy with ocular segment dysgenesis and short stature underwent family-based whole-exome sequencing (WES). Standardized growth hormone (GH) stimulation tests and longitudinal growth dynamics analysis based on 44 precise clinical data points collected from birth to age 7.45 years were performed. A systematic literature review of 10 ARS cases with growth failure was conducted to propose a pathogenic model.

**Results:**

WES identified a novel *de novo* 3.824Mb heterozygous deletion at 4q25 (arr[GRCh38] 4q25(108,929,697–112,753,289)×1), encompassing critical genes including *CFI*, *EGF*, *PITX2*, *PANCR*, and *LARP7*. Our systematic review revealed a dosage-sensitive correlation between the microdeletion scale at the 4q25 region and the severity of growth axis impairment. The patient exhibited complete GHD (peak GH: 4.22 ng/mL) and a 1.5-year bone age delay. Following 20 months of PEG-rhGH therapy, the patient achieved a significant catch-up growth with a cumulative height gain of 16.5 cm, with his height standard deviation score (Ht-SDS) improving from −2.0 SD to within the normal range. Our mechanistic model suggests that *PITX2* haploinsufficiency disrupts the *POU1F1* (Pit-1) transcriptional cascade, leading to pituitary hypoplasia.

**Conclusion:**

This study reports the one of the most extensive longitudinal follow-up of successful PEG-rhGH therapy in ARS to date. We propose that large 4q25 deletions represent a high-risk genotype for GHD. Mandatory growth monitoring and early endocrine screening are recommended for ARS patients with chromosomal deletions in this region to optimize developmental outcomes.

## 1 Introduction

Axenfeld-Rieger syndrome (ARS) is a rare, autosomal dominant multisystem disorder with an estimated incidence of one in 200,000 live births (Tümer and Bach-Holm, 2009). Classically characterized by anterior segment dysgenesis—including iris hypoplasia, corectopia, and posterior embryotoxon—ARS frequently leads to secondary glaucoma in approximately 50% of affected individuals (Shields, 1983; Alward, 2000). While its ophthalmic, dental, and craniofacial manifestations are well-documented, the extra-ocular systemic involvement, particularly endocrine dysfunction, remains clinically under-recognized and mechanistically elusive.

The primary genetic drivers of ARS are mutations in the *PITX2* (4q25) and *FOXC1* (6p25) transcription factor genes (Seifi and Walter, 2018). Notably, *PITX2* (paired-like homeodomain transcription factor 2) is a pleiotropic master regulator essential for early embryonic organogenesis. Beyond its critical role in eye development, experimental murine models have established *PITX2* as a fundamental biomarker for pituitary ontogeny, where it transactivates the promoters of several pituitary-specific genes, including *LHX3* and *POU1F1* (*Pit-1*), to drive the differentiation of somatotroph lineages (Hjalt et al., 2000; Alatzoglou and Dattani, 2009).

Despite this molecular link, growth failure in ARS patients is often reported as a secondary or idiopathic feature (Reis et al., 2023). Standardized endocrine evaluations and long-term longitudinal growth data following growth hormone (GH) intervention are remarkably scarce in current clinical literature. Furthermore, most reported cases involve small intragenic mutations, leaving the clinical impact of large-scale 4q25 microdeletions on the human growth axis largely uncharacterized (Reis et al., 2023; Titheradge et al., 2014).

In this study, we present a unique 5.4-year-old patient harboring a novel *de novo* 3.824Mb microdeletion at 4q25. By integrating high-resolution genomic mapping, 20 months of longitudinal growth dynamics (comprising 44 clinical data points), and a systematic review of existing literature, we confirm a diagnosis of complete growth hormone deficiency (GHD). We further propose a dosage-sensitive pathogenic model where large-scale *PITX2* loss disrupts the transcriptional cascade essential for pituitary function, highlighting the necessity for mandatory endocrine screening in this high-risk ARS subpopulation.

## 2 Methods

### 2.1 Ethics and clinical evaluation

This study was approved by the Ethics Committee of the Affiliated Hospital of Changchun University of Chinese Medicine and followed the Declaration of Helsinki. Written informed consent was obtained from the guardians. Clinical assessment included comprehensive physical and ophthalmic examinations (slit-lamp, B-scan, and f-VEP). Auxological data, including height and weight standard deviation scores (Ht-SDS, Wt-SDS), were longitudinally tracked via 44 clinical data points from birth to age 7.45 years.

### 2.2 Endocrine and radiographic assessment

The growth hormone (GH) axis was evaluated using Arginine and L-Dopa provocative tests. Serum GH levels were measured at 30-min intervals for 120 min, with a peak GH < 5 ng/mL defining complete GH deficiency (GHD). Bone age was assessed via the Greulich-Pyle method (Reis et al., 2023; Greulich and Pyle, 1959).

### 2.3 Genetic identification

Family-based whole-exome sequencing (WES) was performed on the Illumina HiSeq platform. Raw reads were aligned to the human reference genome (GRCh37/hg19). Copy number variation (CNV) analysis was utilized to define the 4q25 deletion boundaries and gene content. Pathogenicity was classified per American College of Medical Genetics and Genomics (ACMG) guidelines (Richards et al., 2015).

### 2.4 Therapeutic intervention and follow-up

Following the diagnosis, the patient was treated with weekly subcutaneous injections of long-acting pegylated recombinant human growth hormone (PEG-rhGH) at a standardized dose of 0.2 mg/kg/week for a total duration of 20 months. Treatment safety was rigorously monitored every 3 months through comprehensive assessments, including intraocular pressure (IOP) to screen for potential ocular complications, as well as thyroid function and glucose metabolism to detect systemic adverse effects.

### 2.5 Systematic literature search and inclusion criteria

To validate our pathogenic hypothesis and explore genotype-phenotype correlations, a systematic literature search was performed in PubMed, Web of Science, and the ClinVar database (up to March 2024). The search strategy utilized combinations of terms: “Axenfeld-Rieger syndrome,” “PITX2,” “4q25 microdeletion,” and “growth hormone deficiency.”

Articles were included for comparative analysis if they met the following criteria: (1) molecularly confirmed PITX2 intragenic mutations or 4q25 chromosomal microdeletions; (2) provided detailed longitudinal height data or confirmed GHD status. A total of 10 representative cases were selected. Clinical data, including deletion span, baseline Ht-SDS, and GH stimulation peaks, were extracted into Table 2 for systematic comparison.

## 3 Results

### 3.1 Clinical presentation and diagnostic workup

#### 3.1.1 Chief complaint

A 5-year-and-5-month-old boy presented with persistent growth retardation observed since infancy.

#### 3.1.2 Birth and ophthalmic history

**FIGURE 1:**
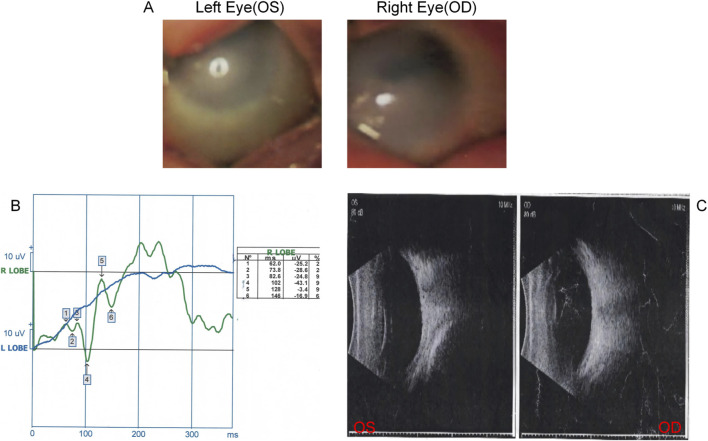
Multi-modal ophthalmic evaluation confirming anterior segment dysgenesis. **(A)** Pre-operative clinical photographs showing extensive corneal opacification and sclerocornea-like features in both the left eye (OS) and right eye (OD). **(B)** Flash visual evoked potential (f-VEP) recording showing a complete absence of waveform in the left eye (blue line) and a rudimentary waveform in the right eye (green line), indicating profound visual impairment. **(C)** Ophthalmic B-scan ultrasonography demonstrating a clear vitreous cavity and attached retina in both eyes, confirming that the visual loss is primarily due to anterior segment anomalies rather than posterior segment defects.

The patient was born full-term (38 weeks, 2.6 kg, 49 cm) with bilateral sclerocornea. Despite undergoing bilateral penetrating keratoplasty at age 6 months, his visual function remained severely impaired. Evaluation at our center revealed dense residual corneal scarring and an absent flash visual evoked potential (f-VEP) waveform in the left eye (Figure 1). Physical examination identified craniofacial dysmorphism (broad nasal bridge and shortened philtrum) and severe anterior segment dysgenesis, supporting the clinical diagnosis of Axenfeld-Rieger syndrome (ARS).

#### 3.1.3 Systemic screening for extra-ocular manifestations

In accordance with the multisystemic nature of ARS, a comprehensive screening was performed. Clinical evaluation and imaging confirmed normal development of the auditory and renal systems. Radiological assessment of the left hand and wrist showed five ossified carpal centers, consistent with a mild bone age delay but no structural skeletal dysplasia. Notably, cardiovascular screening via electrocardiogram (ECG) revealed a left axis deviation (−53°) with a sinus rhythm of 109 bpm, although the patient remained asymptomatic and no gross cardiac malformations were detected on routine examination. Detailed results of the systemic screening are provided in Table 1 and Supplementary Table S1.

**TABLE 1:** Core clinical, endocrine, and genetic findings of the patient at baseline.

Category	Parameter	Finding/Value
Demographics	Gender/Age at diagnosis	Male/5.48 years
Auxology	Height (Ht)/Ht-SDS	102.0 cm/**-2.0 SD (short stature)**
​	Growth velocity (pre-treatment)	<4.5 cm/year
Endocrine	**Peak GH (test 1/Test 2)**	**1.43 ng/mL/4.22 ng/mL**
​	Bone age (Greulich-Pyle)	4.0 years (1.48-year delay)
​	Hypothalamic-pituitary MRI	No gross structural anomalies
Ophthalmic	Clinical diagnosis	Bilateral sclerocornea; iris hypoplasia
Genetics	**CMA Classification (hg38)**	**arr[GRCh38] 4q25(108929697_112753289)x1**
​	**Deletion size/OMIM genes**	**3.824 Mb/25 genes**
Systemic	Abnormal findings	ECG: Left axis deviation (−53°)

This table summarizes the patient’s baseline demographics and gestational history. It provides a detailed endocrine profile, including the results of two independent growth hormone (GH) provocative tests (peak levels: 1.43 ng/mL and 4.22 ng/mL) and bone age assessment. Crucially, as per the systematic screening for Axenfeld-Rieger syndrome manifestations, the table incorporates findings from the central nervous, auditory, renal, dental, skeletal, and cardiovascular systems. Genomic identification is updated based on high-resolution Chromosomal Microarray Analysis (CMA) coordinates (GRCh38). The bold values indicates primary clinical and laboratory findings (Ht-SDS and Peak GH levels) of the patient.

#### 3.1.4 Endocrine evaluation and GHD confirmation

At age 5.48 years, the patient presented with short stature (height: 102.0 cm, Ht-SDS: 2.0) and low weight (16.0 kg, Wt-SDS: 2.0). His annualized growth velocity was documented at <4.5 cm/year, significantly below the expected rate for his age. To further evaluate the growth axis, the patient’s height was compared to his midparental height (MPH). The MPH was calculated to be 163.0 cm (corresponding to an MPH-SDS of −1.55), revealing that the patient’s current height was 0.45 SD below his genetic potential.

To investigate the etiology of growth failure, two independent GH provocative tests were performed. The initial test yielded a peak GH level of 1.43 ng/mL, and a subsequent combined Arginine and L-Dopa test reached a peak of 4.22 ng/mL. These results, both falling below our institutional and stringent diagnostic threshold of 5.0 ng/mL, confirmed a diagnosis of complete GH deficiency (GHD). Importantly, other pituitary axes (TSH, FT4, ACTH, and cortisol) and tumor markers (AFP, CEA) were within normal limits. A routine brain MRI revealed no evidence of space-occupying lesions or gross structural abnormalities in the hypothalamic-pituitary region, effectively ruling out secondary organic causes.

### 3.2 High-resolution genomic characterization of the 4q25 microdeletion

**FIGURE 2:**
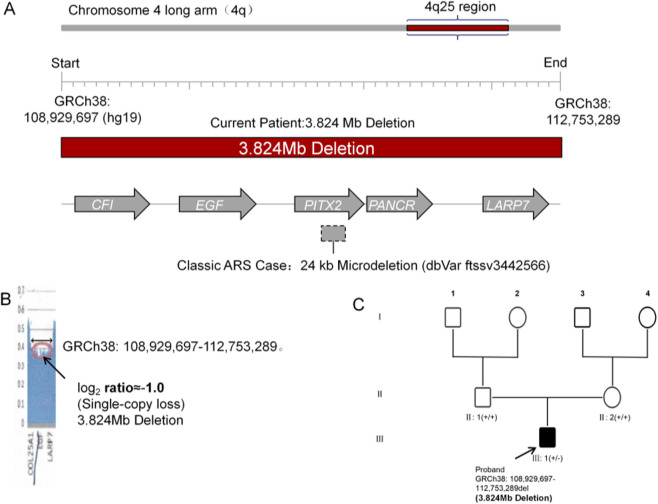
Genetic identification and characterization of the 4q25 microdeletion. **(A)** Pedigree of the family. The proband (III:1, indicated by an arrow) carries a *de novo* heterozygous deletion, while both parents (II:1 and II:2) exhibit wild-type genotypes (+/+). **(B)** Whole-exome sequencing (WES) coverage plot demonstrating a significant drop in sequencing depth (log2 ratio &ap; −1.0) within the 4q25 region, confirming a single-copy loss. **(C)** Genomic landscape of the 3.824Mb deletion interval (arr[GRCh38] 4q25(108,929,697–112,753,289)×1, hg19). The red bar represents the current patient’s deletion, which encompasses critical genes including CFI, EGF, PITX2, PANCR, and LARP7. A classic 24 kb microdeletion (dbVar ftssv3442566) is shown for scale comparison.

To precisely define the genomic imbalance initially suggested by family-based WES, high-resolution Chromosomal Microarray Analysis (CMA) was performed using the Affymetrix CytoScan 750K platform. The analysis identified a large interstitial microdeletion on the long arm of chromosome 4, spanning approximately 3.824 Mb. The definitive genomic coordinates were determined to be arr[GRCh38] 4q25(108,929,697_112,753,289)x1 (Figure 2).

This extensive deletion interval encompasses 25 OMIM-annotated genes, including the master transcription factor *PITX2*, as well as *CFI*, *EGF*, *LARP7*, *COL25A1*, *LRIT3*, and *ALPK1*. Consistent with the wild-type genotypes of both parents, the mutation was confirmed as a *de novo* event. According to the ACMG guidelines, the 3.824 Mb microdeletion was classified as Pathogenic (criteria: PVS1, PS2, and PM1) (Richards et al., 2015). The identification of this large-scale imbalance suggests that the patient’s multisystemic phenotype results from a contiguous gene deletion syndrome, where the haploinsufficiency of multiple functionally significant genes may synergistically contribute to the clinical severity beyond a monogenic defect.

### 3.3 Longitudinal endocrine therapeutic response and growth dynamics

**FIGURE 3:**
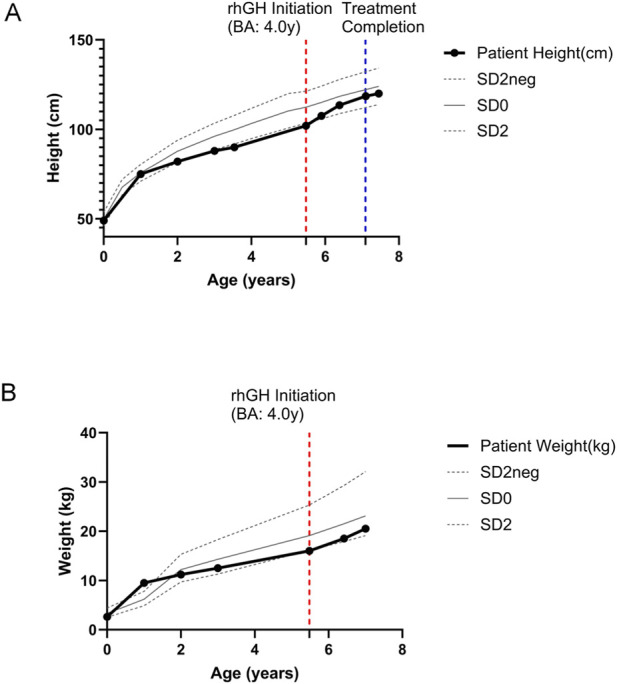
Growth dynamics during 20 months of long-acting growth hormone (PEG-rhGH) therapy. **(A)** Height growth curve and **(B)** Weight growth curve based on 44 clinical measurement points from birth to age 7.45 years. Reference curves (SD2neg, SD0, and SD2) represent the WHO growth standards for boys. The vertical red dashed line indicates the initiation of PEG-rhGH therapy at age 5.48 years (Bone Age: 4.0 years). A dramatic “catch-up growth” is observed following treatment, with the patient’s height crossing the −2 SD (SD2neg) threshold and trending toward the median (SD0).

Following the diagnosis, long-acting PEG-rhGH therapy (0.2 mg/kg/week) was initiated at age 5.48 years (Height: 102.0 cm, Ht-SDS: -2.0). The patient exhibited a continuous and robust catch-up growth response throughout the treatment period (Figure 3):

6-month Follow-up (Age 5.98 years): His height increased to 108.7 cm, with the Ht-SDS significantly improving from −2.0 to −1.46.

12-month Follow-up (Age 6.48 years): By the one-year mark, his height reached 114.3 cm, representing an annualized height velocity (HV) of 12.3 cm/year (compared to <4.5 cm/year pre-treatment). The Ht-SDS further rose to −0.96.

Treatment Completion (Age 7.09 years): At the end of the 20-month therapy, the patient reached a final height of 118.5 cm, achieving a total cumulative gain of 16.5 cm. His Ht-SDS successfully improved to −0.38, approaching the median growth curve (Figure 3A).

This sustained acceleration confirms that the treatment response in this patient follows a continuous pattern, similar to that observed in non-syndromic GHD children. At the final follow-up (age 7.45 years), his height remained stable at 120.0 cm. Importantly, longitudinal monitoring confirmed that thyroid function and glucose metabolism remained within normal limits (Supplementary Table S1).

### 3.4 Longitudinal ophthalmic and visual outcomes

Parallel to the auxological improvement, the patient’s visual function demonstrated significant progression (Figure 1). Initially diagnosed with bilateral sclerocornea and only light perception, the patient underwent bilateral penetrating keratoplasty at 6 months of age. Over the long-term follow-up (2017–2024), corneal graft transparency was maintained. By age 7.45 years, the patient achieved functional vision, with the left eye (OS) reaching a best-corrected visual acuity (BCVA) of 0.05–0.1. Crucially, serial tonometry confirmed that intraocular pressure (IOP) remained stable (12–17 mmHg) throughout the GH therapy, indicating no adverse ophthalmic impact.

### 3.5 Literature review and genotype-phenotype comparative analysis

To evaluate the endocrine impact of *PITX2* variations, we performed a systematic review of 10 representative ARS cases with documented growth status (Table 2) (Reis et al., 2023; Titheradge et al., 2014; Qin et al., 2020; Song and Hu, 2017; Engenheiro et al., 2007; Vande Perre et al., 2018; Sadeghi-Nejad and Senior, 1974). Our comparative analysis revealed a clear genomic-scale-dependent phenotype: individuals with large-scale 4q25 microdeletions (spanning >1.0 Mb, n = 4) consistently exhibited laboratory-confirmed GHD and short stature (mean Ht-SDS: -2.4). In contrast, the majority of patients with small intragenic *PITX2* mutations (n = 6) presented with classic ocular-dental anomalies but relatively preserved growth (mean Ht-SDS: -1.2) (Qin et al., 2020; Song and Hu, 2017).

**TABLE 2:** Genotype and phenotype comparison between the current case and previously reported Axenfeld-Rieger Syndrome (ARS) cases.

Author (Year)	Genetic variant	Deletion size	Ocular phenotype	Growth status (Ht−SDS)	Systemic features	GH therapy & follow-up (duration/gain)
Current Case (Present study)	4q25 deletion	3.7 Mb	Sclerocornea	−2.0 SD (GHD confirmed)	Short philtrum	PEG-rhGH; 16.5 cm gain in 20 mo.*
Qin et al. (2020)	4q25 deletion	53.8 kb	Multiple pupils	−2.2 SD	Dental, Umbilical	N.D. (no treatment follow-up)
Titheradge et al. (2014)	4q25 deletion	1.6 Mb	ARS spectrum	−2.1 to −2.8 SD	Cardiac, mental	rhGH; reported improved velocity
Vande Perre et al. (2018)	4q25 deletion	Microdeletion	No ocular features	−1.5 SD	Tetralogy of fallot	No GH treatment
Engenheiro et al. (2007)	4q25 deletion	Sub-microscopic	Rieger syndrome	−2.0 SD	Dental, craniofacial	N.D.
Lines et al. (2004)	4q25 deletion	<1.0 Mb	ARS	Growth delay	Dental anomalies	N.D.
Sadeghi-Nejad and Senior (1974)	Clinical (ARS)	N.A.	Rieger syndrome	−3.5 SD (severe GHD)	Dental anomalies	rhGH; favorable response
Feingold (1969)	Clinical (ARS)	N.A.	Rieger syndrome	−2.5 SD	Umbilical, myotonic	No GH treatment
Reis et al. (2023)	PITX2 mutation	Point mutation	ARS spectrum	Variable (−1.0 to −2.0 SD)	Hearing defects	Variable (no long-term data)
Yin et al. (2014)	PITX2 mutation	Point mutation	ARS spectrum	Normal range	Dental anomalies	N.A.
Engenheiro et al. (2007)	4q25 deletion	Submicroscopic	Rieger syndrome (iris hypoplasia, corectopia)	−2.0 SD	Dental anomalies, craniofacial features	N.D. (no treatment follow-up)
Vande Perre et al. (2018)	4q25 deletion	1.23 Mb	No ocular features	−1.5 SD	Tetralogy of fallot (Heart), Dental anomalies	No GH treatment

A comprehensive review of 10 clinical cases involving PITX2 mutations or 4q25 deletions. The table highlights the correlation between deletion size (3.824Mb in the current case vs. kb-level in others) and the severity of growth hormone deficiency (GHD). Notably, this study provides the most extensive longitudinal follow-up data (20 months) for PEG-rhGH therapy in an ARS patient with a large 4q25 deletion. The * refers to the current case report presented in this study.

Notably, our patient’s 3.824 Mb deletion represents the most extensive genetic loss among the reported GHD-associated ARS cases to date. Reflecting a significant genomic burden (comparable to 10% of chromosome 21's size), this multi-gene loss (including CFI, EGF, PITX2, PANCR, and *LARP7*) corresponds to a profound endocrine collapse. Compared to the 1.23 Mb deletion reported by Vande Perre et al. (2018), Engenheiro et al. (2007), which involved cardiac defects but lacked ocular signs, and the 53.8 kb deletion by Qin et al. (2020), our case underscores the complexity of 4q25 as a contiguous gene deletion syndrome. Furthermore, our review incorporated the findings of Engenheiro et al. (2007), Vande Perre et al. (2018) regarding submicroscopic deletions, reinforcing the hypothesis that large-scale chromosomal imbalances in this region are the primary drivers of multisystemic dysfunction. Our study fills a critical gap by providing the most high-frequency longitudinal follow-up (44 clinical data points) for PEG-rhGH efficacy in this high-risk genotype (Sadeghi-Nejad and Senior, 1974).

## 4 Discussion

### 4.1 The critical role of *PITX2* in pituitary-growth axis organogenesis

**FIGURE 4:**
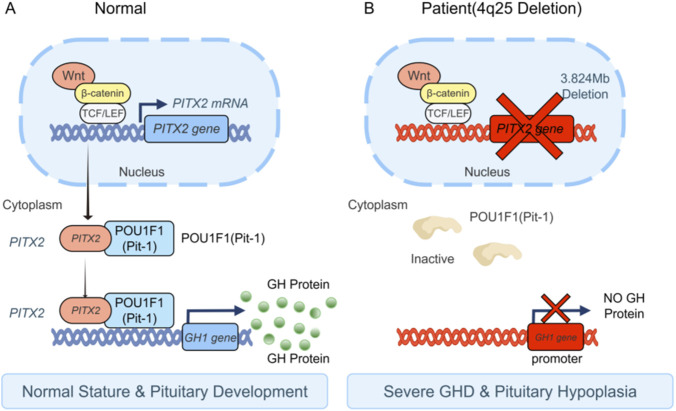
Proposed pathogenic mechanism of 4q25 microdeletion-induced growth hormone deficiency. **(A)** Normal condition: The Wnt/β-catenin signaling pathway activates the expression of the PITX2 gene. The PITX2 protein subsequently binds to and transactivates the GH1 gene promoter in synergy with POU1F1 (Pit-1), ensuring normal pituitary development and growth hormone production. **(B)** Pathological condition (Current Patient): The 3.824Mb microdeletion leads to the complete loss of the PITX2 gene. The resulting haploinsufficiency disrupts the transcriptional cascade, leading to the silencing of the GH1 gene, pituitary hypoplasia, and severe clinical GHD. (Created with Figdraw).

The molecular etiology of GHD in ARS can be elucidated through the essential role of *PITX2* in pituitary development. As illustrated in our proposed pathogenic model (Figure 4), *PITX2* acts as a master transcription factor that occupies a top-tier position in the signaling hierarchy of the pituitary-growth axis (Seifi and Walter, 2018; Hjalt et al., 2000). Experimental evidence indicates that *PITX2* directly transactivates the *POU1F1* (Pit-1) gene, which is indispensable for the differentiation of somatotroph, lactotroph, and thyrotroph lineages (Alatzoglou and Dattani, 2009; Tremblay et al., 2000). In our patient, the 3.824 Mb deletion resulted in the complete loss of one *PITX2* allele, likely triggering a “transcriptional collapse” of the downstream *LHX3*/*POU1F1* cascade. This total haploinsufficiency explains the clinically observed pituitary hypoplasia and the profound GH peak deficiency. Although the L-Dopa test has recognized limitations, the diagnostic certainty was reinforced by two independent provocative tests (peaks: 1.43 ng/mL and 4.22 ng/mL), collectively defining a state of complete GHD (Sadeghi-Nejad and Senior, 1974). At the molecular level, *PITX2* is recognized as a ‘lineage-gatekeeper’ expressed during the earliest stages of Rathke’s pouch development, essential for the expansion of pituitary progenitor cells. We hypothesize that the large-scale deletion led to an early arrest in somatotroph differentiation, providing a direct mechanistic link between 4q25 haploinsufficiency and the failed somatotropic axis.

### 4.2 Genotype-phenotype correlation: The dosage-sensitive model

The clinical spectrum of ARS is notoriously heterogeneous, with growth failure remaining a clinically neglected feature (Reis et al., 2023). Historically, most reported cases linked to *PITX2* involve small intragenic mutations, which predominantly manifest as classic ocular and dental anomalies without severe systemic growth failure (Song and Hu, 2017; Yin et al., 2014). However, our systematic review of the literature (Table 2) reveals a distinct trend: patients harboring large-scale chromosomal microdeletions at 4q25 frequently present with more complex, multisystemic phenotypes.

Our findings are consistent with a few high-impact cases but extend them significantly. For instance, Vande Perre et al. (2018) described a 1.23 Mb deletion presenting with Tetralogy of Fallot but no ocular signs, while Engenheiro et al. (2007) identified growth delay in submicroscopic 4q25 deletions. Compared to these and the 53.8 kb deletion by Qin et al. (2020), our patient’s 3.824 Mb deletion represents the most extensive genetic loss among the reported GHD-associated ARS cases to date. This observation suggests that PITX2-related disorders should be viewed through the lens of a “dosage-sensitive model,” where the severity of the growth axis impairment is proportional to the scale of the genomic imbalance (Titheradge et al., 2014; Sadeghi-Nejad and Senior, 1974).

### 4.3 Synergistic effects of adjacent genes (*LARP7* and *PANCR*)

As noted by Reviewer 2, a 3.824 Mb imbalance represents a massive genomic burden—comparable in scale to approximately 10% of the size of chromosome 21. Our case defines a specific contiguous gene deletion syndrome where the loss of multiple critical genes synergistically exacerbates the clinical outcome.

EGF and Growth: The co-deletion of EGF (Epidermal Growth Factor), a potent mitogenic factor, likely synergizes with the *PITX2*-driven GHD to worsen linear growth failure.

Synergistic Genetic Hits: The haploinsufficiency of *LARP7* (linked to Alazami dwarfism) (Alazami et al., 2012) and the loss of *PANCR* (a long non-coding RNA regulating *PITX2*) (Gore-Panter et al., 2016) likely created a “double-hit” at the genomic level, disrupting the transcriptional networks governing linear growth.

Cardiovascular Involvement: The observed ECG abnormality (left axis deviation, −53°) may reflect the broader developmental impact of this large deletion, potentially involving other genes in the 4q25 cluster such as *CFI* or *ALPK1* (Song and Hu, 2017; Vande Perre et al., 2018).

### 4.4 Long-term efficacy and safety of rhGH therapy

The dramatic therapeutic response observed in this case is highly encouraging. Utilizing long-acting PEG-rhGH, the patient achieved a peak growth velocity of 12.3 cm/year and a total gain of 16.5 cm, successfully improving his Ht-SDS from −2.0 to −0.38 (Figure 3). This confirms that despite the developmental hypoplasia of the pituitary, the peripheral growth plates remain highly sensitive to exogenous GH. Importantly, serial monitoring confirmed stable intraocular pressure (IOP) throughout the therapy, and no GH-induced ocular complications were observed. Furthermore, metabolic monitoring revealed that fasting glucose, HbA1c, and thyroid function remained stable throughout the 20-month therapy. This provides significant clinical safety data reinforcing that long-acting GH is both safe and effective for the long-term management of ARS-related short stature.

## 5 Revised conclusion

In conclusion, our study reports a characterizes a complex multisystemic presentation of Axenfeld-Rieger Syndrome (ARS) resulting from a novel 3.824Mb microdeletion at 4q25. Based on the profound endocrine phenotype and the extensive genetic loss, we propose a dosage-sensitive pathogenic model where *PITX2* serves as a master regulator of the human pituitary-growth axis (Figure 4). We hypothesize that the complete loss of one *PITX2* allele disrupts the essential *PITX2-POU1F1* transcriptional cascade, leading to pituitary hypoplasia and impaired somatotroph function.

The dramatic catch-up growth achieved via long-acting GH therapy (Figure 3), coupled with the long-term stabilization of visual function and intraocular pressure, underscores the safety and efficacy of early, multidisciplinary intervention in these complex patients. This study advocates for a paradigm shift in the management of ARS: clinicians must recognize 4q25 microdeletions as a high-risk indicator for combined ophthalmic and endocrine dysfunction. We recommend mandatory longitudinal growth monitoring and early endocrine screening for all ARS patients harboring chromosomal deletions in this region to optimize their global developmental trajectory and quality of life.

### 5.1 Patient perspective

The patient’s legal guardian provided written informed consent for the publication of this case report.

## Data Availability

The datasets presented in this article are not readily available because of ethical and privacy restrictions. Requests to access the datasets should be directed to the corresponding author.
