# Supplementary material for: Efficacy of long-acting growth hormone in Axenfeld-Rieger syndrome with a novel 3.824 Mb 4q25 deletion: a Case Report and systematic literature review
Source: Front Genet. 2026 Jun 17;17:1856235. doi: 10.3389/fgene.2026.1856235 (PMC13318242; doi:10.3389/fgene.2026.1856235)
Supplement: Supplementary file 1 [file Table1.docx]

| **System / Axis** | **Parameter** | **Result / Range** | **Clinical Significance** |
| --- | --- | --- | --- |
| **Pituitary Axes** | TSH / Free T4 | 1.05–2.16 μIU/mL / Normal | Ruled out CPHD |
|  | LH / FSH / E2 / T / PRL | Within prepubertal ranges | Intact gonadotropic axis |
| **Metabolism** | Fasting Glucose / HbA1c | 3.60–4.48 mmol/L / Stable | No GH-induced diabetes |
|  | 25-OH Vitamin D | 62.4 nmol/L (Normal) | Baseline bone health |
| **Organ Systems** | Hearing (Audiometry) | Normal | No extra-ocular ARS signs |
|  | Renal (Ultrasound) | Normal | No renal hypoplasia |
|  | Dental (Physical exam) | Normal | No microdontia |
|  | Skeletal (X-ray) | Normal structure (5 carpals) | No skeletal dysplasia |
| **Biochemistry** | Liver (AST) | 58–59 U/L (Mild baseline elevation) | Monitored safety |
|  | Renal (BUN / Cr / UA) | 5.3 mmol/L / 20 μmol/L / 235 μmol/L | Normal function |
|  | Cardiac markers (CK-MB/LDH) | 22–105 U/L / 262–299 U/L | Linked to baseline ECG |
| **Tumor Markers** | AFP / CEA | Negative / Normal | Ruled out organic lesions |
| **Urine Routine** | Ketones (KET) / Specific Gravity | Negative / 1.005–1.030 | Stable hydration/metabolism |

**Supplementary Table S1. Expanded systemic screening and longitudinal biochemical parameters.**
